# Patient-reported quality of life (QoL) measurements in adults with multiple long-term conditions: A scoping review protocol

**DOI:** 10.1177/26335565251390804

**Published:** 2025-11-01

**Authors:** Marta Santillo, Laura J. Gray, Hannah M. L. Young, Jonathan A. Batty, Claire Brockett, Vishal R. Aggarwal, Thomas Beaney, Lihua Wu, Sivesh Kamarajah, Nathan Davies, Nia Roberts, Tanya MacKay, Megan A. Kirk

**Affiliations:** 1 Nuffield Department of Primary Care Health Sciences, Medical Sciences Division, 6396 University of Oxford, Oxford, UK; 2 Department of Population Health Sciences, 4488 University of Leicester, Leicester, UK; 3 NIHR Applied Research Collaboration East Midlands, 4488 University of Leicester, Leicester, UK; 4 NIHR Leicester Biomedical Research Centre, 4488 University of Leicester and University Hospitals of Leicester NHS Trust, Leicester, UK; 5 Leicester British Heart Foundation Centre of Research Excellence, 4488 University of Leicester, UK; 6 Leicester Diabetes Centre, College of Life Sciences, 4488 University of Leicester, Leicester, UK; 7 Therapy Department, University of Hospitals of Leicester NHS Trust, Leicester, UK; 8 Leads Institute of Cardiovascular and Metabolic Medicine, School of Medicine, Faculty of Medicine and Health, 4468 University of Leeds, Leeds, UK; 9 Leeds Institute of for Data Analytics, 4468 University of Leeds, Leeds, UK; 10 School of Mechanical, Aerospace and Civil Engineering, University of Sheffield, Sheffield, UK; 11 School of Dentistry, Faculty of Medicine and Health, 4468 University of Leeds, Leeds, UK; 12 The George Institute for Global Health, 4615 Imperial College London, London, UK; 13 School of Nursing, Allied and Public Health, Faculty of Health, Science, Social Care and Education, Kingston University London, London, UK; 14 Department of Applied Health Sciences, 1724 University of Birmingham, Birmingham, UK; 15 Patient and Public Representative, Wales, UK; 16 Bodleian Health Care Libraries, 6396 University of Oxford, Oxford, UK; 17 Head of Research and Involvement, McPin Foundation, London, UK; 18 NIHR Oxford Health Biomedical Research Centre (BRC), Department of Psychiatry, 6396 University of Oxford, Oxford, UK; 19 NIHR Applied Research Collaboration Oxford and Thames Valley, Nuffield Department of Primary Care Health Sciences, 6396 University of Oxford, Oxford, UK

**Keywords:** multiple long-term conditions, multimorbidity, quality of life, patient-reported outcome measures, scoping review

## Abstract

**Objective:**

This scoping review will systematically map the evidence on Patient Reported Outcome Measures (PROMS) used to assess quality of life (QoL) in adults with multiple long-term conditions (MLTC) across all healthcare and community settings.

**Rationale:**

Research on patient-reported QoL in adults with MLTC is limited. Existing measures are mostly generic and may lack sensitivity to the complexity and heterogeneity of MLTC. This review will examine PROMs used in MLTC research, and identify gaps in QoL measurement.

**Inclusion/Exclusion criteria:**

Quantitative, qualitative, or mixed-methods studies, and pre-specified grey literature, reporting QoL tools for adults with any combination of physical and/or mental MLTC will be included. Single conditions, comorbidity, or non-adult populations will be excluded.

**Methods:**

Following established scoping review guidelines, a systematic search strategy, developed with a librarian, will cover fivedatabases (e.g., MEDLINE, Embase, PsychINFO, CINAHL), plus grey literature and citation tracking. English-language publications with no restrictions on geographic location or publication date will be considered. After de-duplication, two reviewers will independently screen citations based on predefined inclusion criteria. Discrepancies will be resolved with a third reviewer. A pre-specified data extraction form to capture qualitative and quantitative data will be pilot tested. An 11-member patient and public advisory group will be established and stakeholder consultation will be conducted. Findings will be summarised using tables, figures and narrative synthesis and disseminated widely for multiple audiences.

**Discussion:**

This review will highlight QoL measurement gaps, inform future development of tailored QoL PROMs for MLTC populations, and contribute to national or global MLTC research priorities.

## Introduction

The prevalence of Multiple Long-Term Conditions (MLTC) is rising due to population ageing, advancements in healthcare, and widening health inequalities, presenting an urgent public health challenge.^1^ In England, over 8 million adults (14.8% of the total population) live with MLTC,^2,3^ defined as the presence of two or more chronic conditions, with wide variation in which constituent conditions may be included.^4,5^ The distribution of MLTC is heterogenous and varies across the life-course with asthma and depression most common in young adults, depression and hypertension most common in mid-life, and cardiometabolic disease and osteoarthritis most common in older adults.^2^

People living with MLTC experience increased healthcare needs, poorer health outcomes, greater treatment burden, and lower satisfaction with healthcare services, all of which diminish health-related quality of life^6,7^ (QoL). QoL, defined by the World Health Organization (WHO), refers to “an individual’s perception of their position in life in the context of the culture and value systems in which they live and in relation to their goals, expectations, standards and concerns”.^8^ Improving QoL measurement has been identified as a high-priority area in MLTC research.^9^ A 2019 meta-analysis^10^ on multimorbidity and QoL found that most studies used generic QoL instruments, with only two studies using a condition-specific scale. Moreover, few clinical trials in MLTC research have evaluated interventions designed to improve QoL in this population, despite a clear need.^11^

Patient Reported Outcome Measures (PROMS) are essential tools to capture patients’ perspectives on QoL encompassing social, emotional, and psychological domains.^12^ However, a 2022 review on medication-related QoL PROMS found most PROMS were developed for single disease conditions, with few validated or tailored to the complexity of MLTC.^13^ Similarly, a 2020 review by MØller et al.^14^, critically assessed the measurement adequacy of six PROMs used in multimorbidity research. The authors focused exclusively on primary care settings and found significant limitations in content validity, development methodology, and psychometric robustness. Notably, none of the PROMs were specifically designed to measure QoL in adults with MLTC.^14^ These reviews have advanced the field of patient-reported QoL measures in MLTC, but highlight the need for an updated and more comprehensive synthesis that addresses a broader setting and the gaps and limitations of prior work, particularly in light of rapid developments in this field.

MLTC disproportionately affect adults from lower socioeconomic backgrounds and ethnically diverse communities who often face additional barriers in healthcare access, discrimination, and social stigma.^15^ MLTC include a range of different combinations of diverse conditions, yet there are patterns of shared experiences across patients with MLTC^16^ making it crucial to understand such patterns. This aligns with the National Institute of Health and Care Research (NIHR) strategic framework and emphasis on person-centred care.^16^ A patient-centred approach focuses on “achieving patients’ targets for life and health while imposing the minimal potential treatment burden on their lives with an empathic and feasible treatment plan”^17^ This scoping review will adopt this focus, moving beyond disease-specific and treatment-burden frameworks.

To date, reviews of QoL in MLTC populations have primarily focused on measurement tools that have not been developed specifically for this group. Prior research has emphasised disease-centric aspects of QoL, such as treatment burden or illness perceptions, without providing a comprehensive, person-centred perspective. Furthermore, widely used QoL measures may not capture the lived experiences or cultural interpretations of well-being among diverse MLTC populations, highlighting a critical measurement gap.

The WHO’s International Classification of Functioning (ICF) provides a holistic framework that helps to identify the gaps in current measures, capturing not only health and disability, but activity, participation, and the influence of environmental factors. This makes it particularly relevant to MLTC, given the complex inter-relationships between chronic conditions, social support and functioning. Although widely used in other chronic disease contexts, it has not been systematically applied in the context of MLTC.^18,19^

To our knowledge, no scoping review has systematically mapped the full range of PROMs used to assess QoL in MLTC populations across healthcare and community settings. Furthermore, no prior review has applied the WHO’s International Classification of Functioning (ICF) framework to systematically identify conceptual gaps in current QoL measurement in MLTC populations. This review seeks to address these limitations by synthesising and evaluating the scope, content, and context of QoL PROMs in MLTC populations.

## Research aim and questions

The primary aim of this scoping review is to systematically map and evaluate the existing literature on patient-reported quality of life (QoL) measures in adults living with MLTC, using the ICF framework to identify conceptual and contextual gaps.

To address this aim, our scoping review will address the following research questions:

1. What patient-reported QoL measures have been used in research involving adults living with MLTC?
2. Are there any patient-reported QoL measures that have been developed specifically for those with MLTC?
3. What domains or components of QoL do the measures identified in Research Question 1 capture, and how do these align with the ICF framework?

## Methods

### Design

This comprehensive scoping review will follow a structured multi-stage process outlined by Arksey and O’Malley^20^ and will also include facilitated PPIE and stakeholder consultation sessions (e.g., patients with MLTC and/or carers, clinicians, service providers) to help inform recommendations on future QoL measurement in MLTC. The proposed scoping review will be conducted in accordance with the JBI methodology for scoping reviews^21^ and will follow the Preferred Reporting Items for Systematic Reviews and Meta-Analyses (PRISMA) reporting guidelines for scoping reviews.^22^ The protocol has been preregistered on Open Science Framework (OSF) (https://doi.org/10.17605/OSF.IO/T97P2).

### Population inclusion and exclusion criteria

This scoping review summarises literature on adults, aged 18 years and older, living with two or more long-term physical and/or mental health conditions. The definition of MLTC follows NIHR guidelines^23^ and refers to the presence of two or more long-term conditions in a single individual, such as: (1) a mental health condition of long duration such as depression, schizophrenia, or dementia, (2) a physical, non-communicable health condition such as cancer, coronary heart disease, or diabetes, and (3) a long-term infectious disease such as HIV. Within this review, populations that are required to have a specific index condition (e.g., COPD) and studies focused on co-morbidity (defined by the presence of a single index condition plus one or more other) will be excluded.

### Concept

This review will include studies that investigate the development, conceptualisation or use of QoL measures, including PROMs, used in and/or specifically designed for adults with MLTC. We are guided by the WHO^8^ QoL definition as, “an individual’s perception of their position in life in the context of the culture and value systems in which they live and in relation to their goals, expectations, standards and concerns.” We recognise that QoL has a wide range of definitions and perspectives so we will also include studies which use any measure of QoL, or where authors have stated that they are focused on or measure QoL.

### Context

This review will include literature across all research and healthcare settings including primary, secondary, and tertiary healthcare settings, community and workplace settings, individual settings, and social care.

### Types of sources

This scoping review will include relevant quantitative, qualitative, mixed-methods, and relevant grey literature. Quantitative studies such as experimental, observational, measurement validation, quasi-experimental study designs will be included. Trial protocols will not be included as they are unlikely to include the level of information required. However, they will be saved to search for relevant published trials that meet inclusion criteria. Qualitative and mixed-methods studies that focus on qualitative data including, but not limited to, designs such as phenomenology, grounded theory, ethnography, qualitative description, action research and feminist research will be included.

### Search strategy

A three-step search strategy will be utilised in this review. First, an initial test search was executed by the lead reviewer (MS) in MEDLINE (PubMed) to identify articles on the topic using initial indexing terms and commonly used terminology in the literature. The words contained in the titles and abstracts of relevant articles, and the index terms used to describe the articles were used to develop a full search strategy. To refine the search strategy, key word identification and revision was informed through multiple rounds of consultation with all co-authors in consultation with a subject specialist librarian (ND) who advised suitable terminology, databases and review strategy. Searches and terms will be tailored for each database with support from the subject specialist librarian. Proposed search terms are listed in Supplemental Appendix I.

Secondly, a full search of all relevant literature will be performed by the subject specialist librarian, NR, and include the following databases.

(1) Medline(OvidSP)[1946-],
(2) Embase(OvidSP)[1974-],
(3) PsycINFO(OvidSP)[1806-],
(4) CINAHL(EBSCOHOst)[1982-],
(5) Science Citation Index and Social Science Citation Index (Web of Science Core Collection)[1900-].

Third, the reference lists of all included sources of evidence, including systematic reviews on closely related topics, search of Google Scholar, and relevant websites, will be examined for additional literature and to ensure comprehensive coverage of the literature. Forward and backward citation searching of included studies and relevant systematic reviews will be conducted using CitationChaser.^24^

To ensure a comprehensive synthesis of evidence not indexed in academic databases, we will include reputable sources of unpublished studies and grey literature, such as reports and government and health care organization documents, in our scoping review. Grey literature sources will be identified through targeted searches of government and non-governmental organisations (NGO) websites, such as domains ending in “.gov.uk” and websites of key international organisations such as the World Health Organisation (WHO). We will conduct structured searches using predefined search terms to locate relevant grey literature aligned with our research questions. To maintain feasibility and relevance, we will screen the first 10 pages of results. Identified documents will be screened for eligibility based on the same inclusion and exclusion criteria applied to peer-reviewed literature, and the grey literature search process will be documented and reported transparently in the final review.

No time limit will be set for when papers were published. There will be no initial language restriction.

### Study selection

Following the search, all identified citations will be collated and uploaded into Covidence (Veritas Health Innovation, Melbourne, Australia) and duplicates removed. Covidence will be used to facilitate the screening progress.

To ensure relevant articles have been accurately identified, 2 independent researchers will pilot test the titles and abstract screening processes against the following criteria: publication discusses an unselected population of individual with MLTC, and Patient reported QoL measures; studied adults (aged ≥ 18 years); uses a quantitative, qualitative, experimental, observational, or mixed methods design; excluding conference abstracts, editorials or opinion papers. Following this, two or more independent reviewers will conduct the screening of remaining titles and abstracts for assessment against the inclusion criteria listed here.

The full text of selected citations will be independently assessed in detail against the inclusion criteria by two or more reviewers. All papers in languages other than English will be excluded at this stage, as there are no available resources for translation within the time frame of the scoping review. Reasons for exclusion of sources of evidence at full text that do not meet the inclusion criteria will be recorded and reported. Any disagreements that arise between the reviewers at each stage of the selection process will be resolved through discussion or with recourse to an additional reviewer as required. The results of the search and the study inclusion process will be presented in full in the final scoping review and presented in a PRISMA flow diagram.^25^

### Data extraction

Data will be extracted from the included papers by two or more independent reviewers using a data extraction tool developed by the reviewers in consultation with the PAG members (see Supplemental Appendix II). To ensure that the data is extracted accurately, two independent reviewers will pilot test the data extraction tool prior to extraction. Modifications will be detailed in the scoping review. The data to be extracted will include: author(s), year of publication, journal, country of origin, aim of the study, research type, research design, how data on MLTC was extracted, definition of MLTC, whether mental illness was included in the MLTC, average number of conditions reported, study sample size, participants demographics, how many and which QoL measurement tools were used, information on QoL measurement tool validation. Measurement validity will be in two steps. As a first step we will conduct a separate search to retrieve the original validation paper for each QoL measure. As a second step we will assess whether each QoL measure was validated in a population with MLTC. Any disagreements that arise between the reviewers will be resolved through discussion, or with an additional reviewer/s. If appropriate, authors of papers will be contacted to request missing or additional data, where required.

#### ICF coding

**Figure 1.**
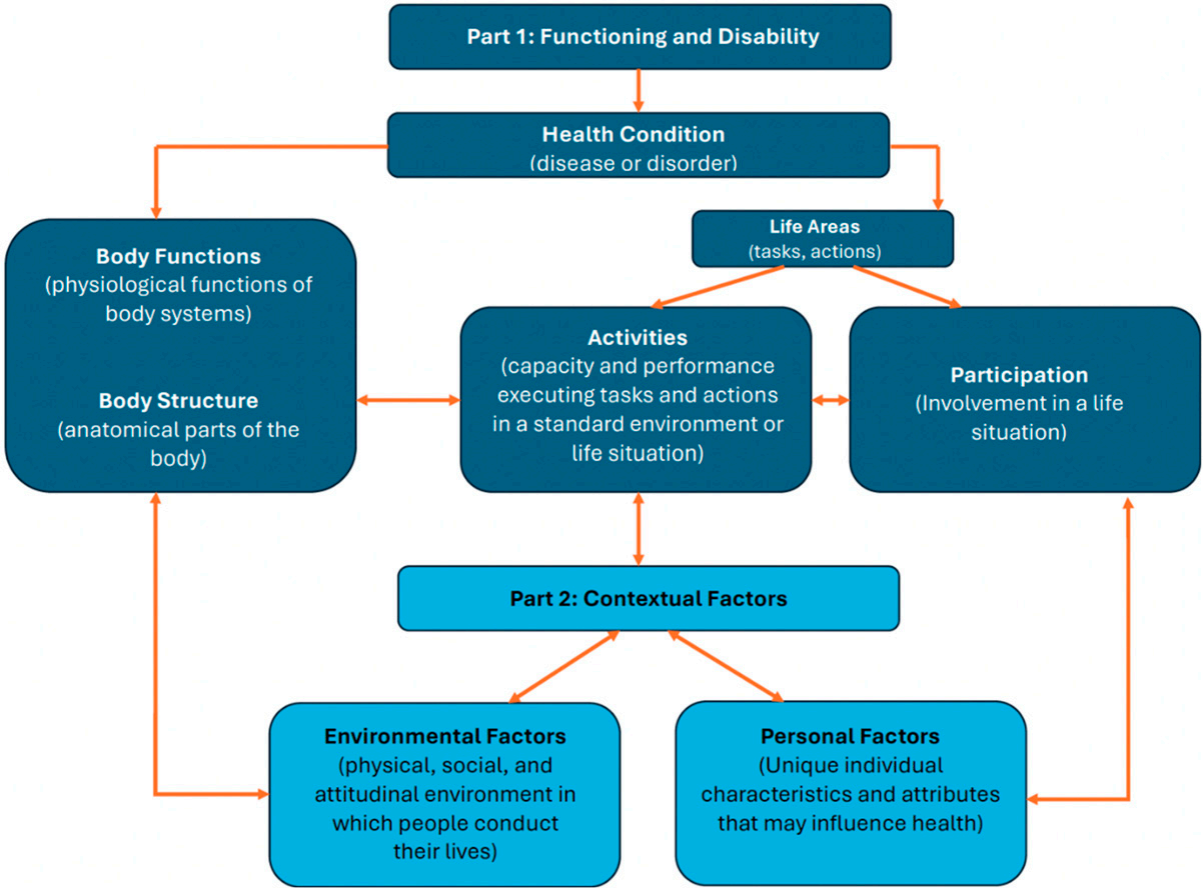
Components of the International Classification of Functioning Disability and Health adapted from WHO (2001)^8^.

Once the QoL measures used for MLTC within the eligible studies (RQ1) have been extracted from the full-text papers retrieved; to address the third research question (RQ3) a second stage of extraction will take place to identify the components of quality of life included within each of these measures. The components within each QoL measure will be charted using the WHO’s International Classification of Functioning Disability and Health (ICF).^26,27^ The ICF is a framework for describing and measuring health, disability, and functioning by considering body functions, activities, participation, and environmental factors^8^. Figure 1 outlines the components included within the ICF framework.

The ICF will provide a comprehensive conceptual framework for categorising and understanding the components of quality of life which have been measured within existing quality of life measures, and consequently also enable us to identify any gaps in measurement.^26,27^ To complete the ICF extraction, two reviewers will use published linking rules to map outcomes to the ICF.^8,27^ For each QoL measure, meaningful concepts for each component within the measure will be identified and linked to the most relevant category of the ICF, using the ICF browser.^28^ Where this is unclear, an interpretative approach will be used, using supporting literature that describes the conceptualisation and development of the measure^8^ . Agreement on these interpretations will be reached via discussion, with recourse to a third reviewer where required. Components of the quality of life measure which do not fit within these categories will still be recorded and summarised within the review.

### Data analysis and presentation

Data extracted via Covidence will be presented in tabular format to summarise the concept of this review. Tables will present characteristics of the studies, the definition of MLTC, patient-reported QoL measures (including name of the QoL measure and whether it was designed specifically for patients with MLTC). The tables will be accompanied by a narrative summary of measures of QoL, which will allow for new understanding of the data. Quantitative studies will be synthesised through a descriptive and mapping approach in accordance with the JBI scoping review guidelines. The synthesis will involve charting key data from each study. Quantitative findings will be collated and summarised using descriptive statistics. We will conduct a thematic synthesis of the qualitative studies that meet inclusion criteria allowing us to synthesise results and new concepts transparently based on best practices from prior research.^29^ Quantitative and qualitative data relating to each research question will be integrated following guidelines on writing and evaluating mixed-methods research.^30^ Our Patient Advisory Group (PAG) will support the categorisation and interpretation of data identified from the analysis. This plan will be refined towards the end of our review.

### PPI engagement and evidence map creation

Our Patient and Public Involvement and Engagement (PPIE) strategy follows the UK standards for public involvement.^31^ Our PAG of 11 members will support the scoping review through its multiple stages: inclusion and exclusion criteria, screening of title and abstracts, full text screening, data extraction, and synthesis. As part of our commitment to PPIE development, one PAG member attended the JBI Scoping Review training course in March 2025, and will actively apply their learning by working with the co-authors to screen relevant literature to be included in the review. Plans to support data extraction training are in progress with the aim to include the PAG member as a co-author on the final manuscript. We aim for the PPI members to represent diversity in terms of underlying conditions, geographic region, age, gender, ethnicity and socioeconomic background. Once the review is complete, we will present the results to the PAG and will discuss their views and interpretations around relevance of the concepts related to quality of life within the existing measures identified in the review and which groups the measures were developed for. PPIE members will also support us in identifying which concepts might not be fully addressed or adequately captured within the existing tools. Finally, through PPIE discussions we aim to identify which are the priorities for future concepts to capture quality of life.

The research team, PPI members and a creative agency, will create an evidence map, which will present the review findings in a user-friendly format. Specifically, during a facilitated co-design meeting, we will co-design visual storytelling media to disseminate the findings of the review to as wide an audience as possible.

## Conclusions

This scoping review will build on earlier work by offering a broader, more inclusive synthesis of QoL measurement in adults living with MLTC. It aims to identify existing PROMs, examine the components of QoL they capture, and systematically categorise these using the WHO ICF framework. Unlike previous reviews, which focused narrowly on psychometric adequacy in limited settings, this review maps the full landscape of PROMs across diverse healthcare and community contexts and integrates patient and public perspectives in the design, analysis and interpretation of results. By highlighting the key gaps in the evidence base, this review will inform future research priorities and assess the need for developing a new, personalised and holistic QoL measure tailored to the needs of MLTC populations.

## Supplemental Material

Supplemental Material - Patient-reported quality of life (QoL) measurements in adults with multiple long-term conditions: A scoping review protocol Supplemental Material for Patient-reported quality of life (QoL) measurements in adults with multiple long-term conditions: A scoping review protocol by Santillo Marta, Gray Laura J., Young Hannah M.L., Batty Jonathan A., Brockett Claire, Aggarwal Vishal R., Beaney Thomas, Wu Lihua, Kamarajah Sivesh, Davies Nathan, Roberts Nia, MacKay Tanya, Kirk Megan A. in Journal of Multimorbidity and Comorbidity.

## Data Availability

This scoping review is based on previously published literature and does not involve the collection of new empirical data. All materials underlying the findings, including template data collection forms, data extracted from included studies, search strategies, and data used for analyses will be made available in the published review as online supplementary material, or linked to OSF (https://doi.org/10.17605/OSF.IO/T97P2) to ensure transparency and reproducibility in line with best practices of evidence synthesis. Any additional reasonable requests can be made to the corresponding author.*
