# Supplementary material for: Patient-reported quality of life (QoL) measurements in adults with multiple long-term conditions: A scoping review protocol
Source: J Multimorb Comorb. 2025 Nov 1;15:26335565251390804. doi: 10.1177/26335565251390804 (PMC12580504; doi:10.1177/26335565251390804)
Supplement: Supplemental Material - Patient-reported quality of life (QoL) measurements in adults with multiple long-term conditions: A scoping review protocol [file sj-pdf-1-cob-10.1177_26335565251390804.pdf]

Supplementary Material

Appendix I: Example Search strategy

Search conducted on Medline

April/2025

	<u>Medline (Ovid MEDLINE® Epub Ahead of Print, In-Process & Other Non-Indexed Citations, Ovid MEDLINE® Daily and Ovid MEDLINE®) 1946 to present</u>	
1	exp Multimorbidity/ or syndemic/	4312
2	(multimorbid* or multidisease* or multicondition* or multipatholog*).ti,ab,kf.	11969
3	((multiple or multi) adj3 (morbid* or comorbid* or co-morbid* or ill* or disease* or condition* or syndrom* or disorder* or patholog*)).ti,ab,kf.	103975
4	(multiple adj (health or chronic or longterm or long-term)).ti,ab,kf.	8052
5	(polymorbid* or poly-morbid* or plurimorbid* or pluri-morbid* or polypatholog* or poly-patholog* or pluripatholog* or pluri-patholog*).ti,ab,kf.	835
6	mltc?.ti,ab,kf.	489
7	1 or 2 or 3 or 5 or 6	116042
8	exp "Quality of Life"/	304189
9	(quality of life or QOL or life quality or hrqol or hrql or hr-ql).ti,ab,kf.	458829
10	(wellbeing or well-being).ti,ab,kf.	179325
11	8 or 9 or 10	667786
12	(patient reported outcome measur* or PROM?).ti,ab,kf,jw.	22655
13	(index or indices or instrument or instruments or measure or measures or questionnaire or questionnaires or profile or profiles or scale or scales or score or scores or status or survey or surveys).ti,ab,kf.	6982269
14	12 or 13	6984765
15	7 and 11 and 14	3637
16	limit 15 to english language	3473

Appendix II: Data extraction instrument

**Item**	**Comments/Instructions**
**Last Name of 1st Author (e.g., Santillo)**	To quickly identify the study citation, input the last name of the 1st author only (do not include the full author list).
**Year of publication**	The publication year (e.g., 2023)
**Title**	Copy and paste the article title for quick access.
**Journal where the article was published**	Include the name of the Journal (e.g., Journal of Comorbidity and Multimorbidity)
**Country/countries where the study was conducted (*Methods*)**	Identify the country that the study was conducted in (e.g., USA) from the list. Write the country in "Other" text box if not in the list. If the study was conducted in more than one country select "Other" and write the countries in the text box (e.g., Germany and Denmark)
**Aims of the study *(at the end of the Introduction)***	Usually located at the end of the introduction.<br>1. Development of a QoL Measurement tool (creation of the tool through steps like defining concepts and generating items);<br>2. Validation (the process of testing and ensuring an questionnaire accurately measures what it's intended to measure and produces reliable results) of a new QoL measure/questionnaire<br>3. Evaluate relationships (association, connection) between MLTCs and QoL<br>4. Evaluate the effect of an intervention on QoL in MLTCs (e.g., exercise program for MLTCs and QoL is measured before and after the intervention)<br>5. Other: any other study aim (e.g., to understand barriers and facilitators of QoL through interviews)
**Research type (*in Methods*)**<br>1. Quantitative<br>2. Qualitative<br>3. Mixed-methods	Indicate the research type found in the methods section. Quantitative - focused on numbers, measurable numeric data, statistical analysis (e.g., correlations, regression). Qualitative - explore thoughts, perspectives,

	experiences and is subjective, thematic, non-numerical (e.g., interviews, focus groups). Mixed-methods - combines quantitative and qualitative in one study (numbers and meaning) – e.g., survey measuring QoL and in-depth interviews to understand QoL perspectives.
**Research design** *(plan on how to answer the research questions)* ***(in Methods)***<br>1. Randomised controlled trial<br>2. Non-randomised experimental study<br>3. Cohort study<br>4. Cross sectional study<br>5. Case control study<br>6. Psychometric (Questionnaire) Validation<br>7. Qualitative interviews/focus group study<br>8. Case series<br>9. Case report<br>10. Clinical prediction rule<br>11. Economic evaluation<br>12. Process evaluation<br>13. Delphi study<br>14. Other	Generally located in the methods section, please indicate the specific study design.
**Settings** *(where the study was conducted/participants were recruited)*<br>1. General Community Setting<br>2. Primary Care<br>3. Secondary Care<br>4. Tertiary Care<br>5. Long-Term Care/Assisted Living<br>6. Home Setting<br>7. Mental Health/Psychiatric Setting	Study setting describes where the care is delivered or where the study takes place.<br>1) General Community Setting:<br>Public health or population-based studies.<br>Examples: schools, workplaces, community centres, mobile health clinics, outreach programs useful for preventive health, behavioural research, health education.<br>2) Primary Care: The first point of contact in

8. Research/Academic Setting<br><br>9. Rehabilitation Setting	the healthcare system. Examples: Family doctors, general practitioners (GPs), community clinics, nurse practitioners. Primary focus is on preventive care, health promotion, diagnosis and treatment of common conditions, chronic disease management.<br><br>3) Secondary Care: Specialised medical services, usually by referral from primary care.<br><br>Examples: Hospital outpatient clinics, cardiologist clinics, orthopaedics, etc. specialists (cardiologists, dermatologists, etc.), short-term hospitalisations. More complex medical issues requiring specialised knowledge or equipment.<br><br>4) Tertiary Care: Highly specialised consultative care, usually in large hospitals or academic medical centres.<br><br>Examples: Neurosurgery, cancer treatment centers, transplant units, advanced cardiac care. Complex procedures and advanced treatments not widely available.<br><br>5) Long-Term Care (LTC) or Assisted living; (e.g., Nursing Home): Services that meet health or personal care needs of patients/residents over an extended period.<br><br>Examples include Nursing homes, assisted living, rehabilitation facilities, home care services for residents/patients with chronic illness, disability, or frailty support; daily living assistance; palliative or end-of-life care.<br><br>6) Home Setting: Care provided in patient's own home. Home visits, Telehealth, home-based physical or mental therapy.<br><br>7) Mental Health/Psychiatric Setting: Dedicated facilities or units for mental health care such as psychiatric hospitals, inpatient mental health units, outpatient counselling services.<br><br>Populations with clinical depression, anxiety,

	bipolar, psychosis, addiction.<br><br>8) Research/Academic Setting: Clinical research facility (CRF), university medical centres, research laboratories, clinical trial unit or academic institutions. Usually have controlled environments designed specifically for experimental treatments, early-phase trials, translational medicine.<br><br>9) Rehabilitation Setting: Main focus is on recovery and functional improvement (not medical diagnosis). Involves physiotherapists, occupational therapists, speech-language pathologists, rehab doctors. Examples include stroke rehab centres, spinal cord injury rehab centres, cardiac or pulmonary (lung) rehab centres, physical rehabilitation centres. Long-term functional support, multi-disciplinary.
**How were data on MLTC extracted (*selected and retrieved*): in methods**<br><br>1. Patient medical records<br>2. Self-reported by participant<br>3. Not reported<br>4. Other (specify: e.g., self-reported by nurse/GP)	Our inclusion criteria is adults 18+ with MLTCs. Indicate how MLTCs were identified/determined in the study. Patient medical records (electronic medical records, GP records) or self-reported (e.g., participant completed questionnaire); Not reported in the study (no mention); Other: Self-reported by the GP/Nurse
**Definition of MLTC used: in methods or results**<br><br>1. 2 or more conditions<br>2. 3 or more conditions<br>3. Other:	If the requirement was only based on Number of conditions, select the appropriate response (2 or more, or 3 or more). Some studies also included a medication prescription requirement along with the number of conditions. For these, select "OTHER" and provide the details. For example, "2 or more conditions AND a minimum of 4 medications on repeat."
**Was mental illness (e.g., depression, anxiety, bipolar) included as one of the possible MLTC conditions in the study?**<br><br>**1. Yes**<br>**2. No**<br>**3. Did not report**	We want to understand how many studies included mental illness (e.g., depression, anxiety, bipolar, schizophrenia) as one of the possible MLTC conditions. Review methods and results (participant demographics). Yes - mental illness was considered one of the MLTC conditions; No - mental illness was not one of the possible MLTC conditions; Did not report - there was no information on MLTC condition types in the manuscript.

**Average number of conditions reported (results/table) – Specify if Mean or median**	What was the average number of MLTC conditions reported for the study sample (located in results/table). Write either Mean = 3.14 or Median = 3; If categorical, write "Categorical"; Use NR = not reported if study did not report.
**Study Sample Size**	Indicate the whole number of observations or individuals included in the study or experiment. If the study reports intervention and control sample sizes separately, add them together to get the overall sample size. Use numerics (e.g., 10234)
**Age (mean, SD) (results/table)**	Report the mean age (SD) of the study sample. Format should look like: 75.4(5.4)<br>Information is found in the results section in a baseline table or the first paragraph. If the study is an intervention with 2 arms, the intervention and control group age(SD) may be reported separately, you will have to calculate the combined mean(SD) by locating the sample size (n) of each arm, mean age, and SD for each arm. There is a free calculator here (or speak with Megan): https://www.statstodo.com/CombineMeansSDs.php
**% Female (results/table)**	Located in the results section. If there are 2 groups reported separately, you will need to calculate the average percentage. If the % male is reported, calculate 100% - male% = % female. Use NR = Not Reported if the study did not report. Use numeric values only: 64.5
**% White ethnicity (results/baseline table)**	Located in the results section baseline table. If there are 2 groups, you will need to calculate the average percentage. If the % non-White is reported, calculate 100% - non-White% = % White. Use NR = Not Reported if the study did not report.
**Were other ethnic categories of the sample reported?**	Yes/No
**If yes, what were the categories that were reported?**	Refer to table 1 (baseline table) or results section for ethnic categories if reported. List them. Can be various categories, but list as the author reported them (e.g., White, Black,

	Asian, Mixed-Race)
***ProEDI list for all - https://www.trialforge.org/wp-content/uploads/2024/05/PRO-EDI-ParticipantsTableShort_PrintVersion_v1_22.03.2024.pdf**<br><br>**Binary (yes, no) response on whether it was reported or not, for the following:**<br><br>**- Gender identity (e.g., man, woman, transgender, non-binary) (yes/no)**<br><br>**- Sexual identity (e.g., bisexual, gay, lesbian, asexual, pansexual, homosexual, heterosexual, etc) (yes/no)**<br><br>**- Race, ethnicity, ancestry (yes/no)**<br><br>**- Socioeconomic status (SES) (yes/no)**<br><br>**- Level of education (e.g., mean or median years or categories of grades completed) (Yes/No)**<br><br>**- Neurodiversity characteristics (e.g., ADHD, Autism/ASD, hyperactivity) (yes/no)**<br><br>**- Physical/Intellectual Disability (Yes/No)**<br><br>**- Marital status (Yes/No)**<br><br>**- Living arrangement/social support (Yes/No)**<br><br>**- Was the tool originally developed in non-English language? (Li suggestion)**	**Gender:** Simple yes/no response. Gender identity could include response items in any combination of the following: man, woman, transgender, non-binary.<br><br>**Sexual orientation/identity:** Simple yes/no response. Sexual orientation/identity could include many response items in any combination. If the author reported any of the following for the study sample: bisexual, heterosexual, pansexual, asexual, lesbian, gay, queer, etc, select yes.<br><br>**Racial/Ethnic Ancestry:** Simple yes/no response. Racial/ethnic ancestry categories can vary by study authors. This is different than ethnicity and refers to an individual's lineage/heritage asking questions about their ancestral identity. A person could be "mixed-race" ethnicity; they could have African American and Polish ancestry - was this asked or reported beyond ethnicity category?<br><br>**Marital Status:** Refer to results and baseline table. Responses could be any combination of: single, widowed, married, divorced, common-law, etc. Yes/No response.<br><br>**Living Arrangement/Social Support:** Results section, baseline table. Social connection captured via living arrangements (e.g., alone, partner, family members, assisted living, etc.) or reporting social support (e.g., social contact, friends, social engagement). Yes/No.<br><br>**SES:** Response items to measure socioeconomic status (SES) vary by study and typically include questions about parental education, occupation, class/status, and income, as well as questions about the individual's own occupation, education, income, and assets. If SES was reported in any capacity, indicate yes.<br><br>**Education Level:** Did authors report mean or median years of education together with an indication of spread such as range or standard deviation? Alternatively, categories of grades, degree level completed could be reported as a

	number and percentage.<br><br>**Neurodiversity:** Neurodiversity réponse categories can vary. Any combination of neurodivergence, ADHD, autism (ASD), dyslexia, dyspraxia, dyscalculia, Tourette's syndrome etc. Indicate if the authors assessed and reported this in the study.<br><br>**Disability:** Physical disability (vision, hearing, mobility impairment) or intellectual disability (or ID) is a term used when a person has certain limitations in cognitive functioning and skills, including conceptual, social and practical skills, such as language, social and self-care skills.<br><br>Responses: 1) Yes, physical disabilities only, 2) Yes, intellectual disabilities only, 3) Yes, both types of disabilities reported, 4) Yes, did not specific (e.g., “disability”), 5) No, none reported
**Was Patient and Public Involvement (PPI), lived-experience perspectives, or patient advisory groups a part of this specific study?**<br><br>**Yes/No**	as there consultation with PPIE for this manuscript and/or study? There is generally a PPI paragraph in the methods.
**How many QoL measurement tools were used in the study?**<br><br>**1. 1**<br>**2. 2**<br>**3. 3**<br>**4. 4**<br>**5. Other (>4)**	Specify the number of QoL measures used in the study. For example, some studies used EQ-5D and EQ-VAS, which would be 2 QoL tools.
**Was/Were any of the QoL measurement tools originally developed in a non-English language (e.g., German, Mandarin)?**<br><br>**Yes**<br><br>**No**	Was the original tool developed in a non-English language (e.g., German, Mandarin, others) and has since been translated to English? Yes/No<br><br>**Example:** MMQ was originally developed in Dutch, and has since been translated to English.
**Was/were any of the QoL measurement tools translated from English to a non-English Language to tailor to the target sample (e.g., Chinese version)?**	Did the authors use a translated version from English to the language of the country the study was conducted in? Example: Chinese-version of SF-12; Italian-version of SF-36. Located in methods section whether the tool was translated. May also need to source the

	protocol paper if cited.<br><br>If study conducted in a non-English country and do not specify, indicate NR not reported.
**Name of the QoL measure (methods/results)**<br><br>1. SF-8 (a brief 8-item version of the widely used generic questionnaire that assess an individual's health-related quality of life measuring physical and mental components)<br>2. SF-12 (*a widely used 12-item questionnaire that assesses an individual's health-related quality of life by measuring both physical and mental health components)*<br>3. SF-36 (*a widely used questionnaire for assessing health-related quality of life. It measures eight health domains: physical functioning, role-physical, bodily pain, general health, vitality, social functioning, role-emotional, and mental health*)<br>4. EQ-5D (*a questionnaire for measuring health-related quality of life. It assesses health along five dimensions: Mobility, Self-care, Usual activities, Pain/discomfort, and Anxiety/depression*)<br>5. EQ-5D-3L<br>6. EQ-5D-5L<br>7. EQ-VAS<br>8. WHOQOL-BREF<br>9. Quality of Life Scale (QOLS)<br>10. Assessment of Quality of Life (AQoL-4D)<br>11. PROMIS Patient-reported Outcomes Measurement Information System – Global Health Scale<br>12. Other (please specify name of tool and short description)	Identify the specific name(s) of the QoL assessment tool(s) used to measure QoL in the study. Select all that apply (some may use more than one). Choose from the list of popular validated QoL tools.<br><br>If study generated, select "Other" - provide the name, acronym of the tool and short description like above examples. Example format: Kirk Quality of Life (KQoL) questionnaire. A 3-item questionnaire to measure health, social function, and life satisfaction.<br><br>If the version is an updated version of one on the list, put in "other" – SF-12v2 or EQ-5D-5L.<br><br>1. SF-12 (a widely used 12-item questionnaire that assesses an individual's health-related quality of life by measuring both physical and mental health components)<br><br>2. SF-36 (a widely used questionnaire for assessing health-related quality of life. It measures eight health domains: physical functioning, role-physical, bodily pain, general health, vitality, social functioning, role-emotional, and mental health)<br><br>3. EQ-5D (a questionnaire for measuring health-related quality of life. It assesses health along five dimensions: Mobility, Self-care, Usual activities, Pain/discomfort, and Anxiety/depression)
**Did the authors cite the main QoL measurement tool validation or development paper?**	Check methods and reference list for whether the authors cited the QoL tool main validation or development paper. Choose

**Yes/No/Other**	"Other" if the study being extracted is the validation/development paper and say so (e.g., This is the validation paper for MMQ)
**Provide the citation or URL link to the main validation paper.**	Authors may cite this in the methods section and the references list. Copy and paste the citation from the reference list, or search Google Scholar for the URL link. Provide full reference details so we can access the paper efficiently. Provide details of the main validation paper citation or link for the QoL tool. Most popular QoL tools have a main validation paper and it may need to be searched. Put NR (Not Reported) if the tool was study-generated without validation.
**What was the target population for whom the measure has been developed?**<br><br>**1. General healthy adult population (generic)**<br>**2. Adults with a single index condition (e.g., cancer, stroke)**<br>**3. Adults with >2 conditions (MLTCs)**<br>**4. Other**	Reviewing the main validation paper, identify the target population for whom the QoL measure has been developed.<br><br>Developed for generic use (generally healthy adults); Single-index condition (Adults with Multiple Sclerosis); Comorbidity (Adults with 2 co-existing conditions like diabetes and hypertension); Adults with MLTCs (>2 conditions)<br><br>Select other if the tools was developed in any other target population (e.g., Older adults only (>65 years), Children, etc.)
**If the QoL tool was a translated version (Chinese SF-12) of the original, what was the target population(s) for who the translated QoL measure was validated in?**<br><br>**1. General healthy adult population (generic)**<br>**2. Adults with a single index condition (e.g., cancer, stroke)**<br>**3. Adults with >2 conditions (MLTCs)**<br>**4. Other**	If the study used a translated version, did they cite the validation paper? Go to the validation paper they cited and identify the population in which the translated QoL tool was validated.
**Any comments, issues, themes, or concerns with the extraction tool or study?**	Free text to include comments, concerns, issues with extraction for this study. These can help guide discussion during consensus or team meetings.<br><br>Examples: You may wish to report exclusion criteria (e.g., those with vision impairment, dementia) to start to see a theme.
